# Depression and Anxiety Change from Adolescence to Adulthood in Individuals with and without Language Impairment

**DOI:** 10.1371/journal.pone.0156678

**Published:** 2016-07-12

**Authors:** Nicola Botting, Umar Toseeb, Andrew Pickles, Kevin Durkin, Gina Conti-Ramsden

**Affiliations:** 1 Language and Communication Science, City University, London, United Kingdom; 2 Department of Psychology, Manchester Metropolitan University, Manchester, United Kingdom; 3 Institute of Psychiatry, Psychology and Neuroscience, King’s College London, London, United Kingdom; 4 School of Psychological Sciences and Health, University of Strathclyde, Glasgow, United Kingdom; 5 School of Psychological Sciences, The University of Manchester, Manchester, United Kingdom; University of Oxford, UNITED KINGDOM

## Abstract

This prospective longitudinal study aims to determine patterns and predictors of change in depression and anxiety from adolescence to adulthood in individuals with language impairment (LI). Individuals with LI originally recruited at age 7 years and a comparison group of age-matched peers (AMPs) were followed from adolescence (16 years) to adulthood (24 years). We determine patterns of change in depression and anxiety using the Child Manifest Anxiety Scale-Revised (CMAS-R) and Short Moods and Feelings Questionnaire (SMFQ). In addition to examining associations with gender, verbal and nonverbal skills, we use a time-varying variable to investigate relationships between depression and anxiety symptoms and transitions in educational/employment circumstances. The results show that anxiety was higher in participants with LI than age matched peers and remained so from adolescence to adulthood. Individuals with LI had higher levels of depression symptoms than did AMPs at 16 years. Levels in those with LI decreased post-compulsory schooling but rose again by 24 years of age. Those who left compulsory school provision (regardless of school type) for more choice-driven college but who were not in full-time employment or study by 24 years of age were more likely to show this depression pathway. Verbal and nonverbal skills were not predictive of this pattern of depression over time. The typical female vulnerability for depression and anxiety was observed for AMPs but not for individuals with LI. These findings have implications for service provision, career/employment advice and support for individuals with a history of LI during different transitions from adolescence to adulthood.

## Introduction

Language impairment (LI) is a neurodevelopmental disorder that affects around 7% of the population and which can take different forms, with either expressive language or both expressive and receptive skills being affected [1]. LI is more common, but much less researched than autism [2]. One reason for this may be the traditional conceptualization that LI is an early childhood disorder that resolves at a young age. However, at least 50% of those initially diagnosed with LI continue to have long-term difficulties with language and communication [3–7].

A link between mental health problems and language impairment has been clearly established in childhood and adolescence [8–12], with anxiety and depression being particularly evident in adolescence in both community and referred samples [8,13]. In contrast, little is known regarding mental health correlates of LI in later adolescence and adulthood, though what we do know indicates a potentially changing picture. A few small-scale studies have reported elevated levels of depression and anxiety symptoms [5,14–16]. Yet, other investigators have found mental health difficulties to decrease among individuals with LI in young adulthood, albeit in a less affected sample with a history of mild/moderate childhood LI [17]. In a large longitudinal cohort, Conti-Ramsden and Botting [13] observed significant differences in anxiety and depression at 16 years between adolescents with LI when compared with their age-matched peers (AMPs). Following the end of compulsory schooling, however, the symptoms in the LI cohort lessened, particularly in depression, converging with those observed for their AMPs [18].

The changing picture from childhood to adolescence and young adulthood may be due to the varying impact of LI at different stages of development and/or to changes in environmental contexts. The impact of LI may be stronger in childhood and adolescence, when language plays a key role in learning, social interaction and emotional regulations. It is known that language continues to develop into adolescence in people with LI where development may begin to plateau for those with typical development [19]. Gains in language and the development of compensatory strategies may have a positive impact on emotional adaptation in early adulthood. Previous research, however, has not found convincing links between mental health difficulties and the severity of LI [10,13,20]. Similarly, evidence of a strong relationship between nonverbal abilities and mental health in adolescents with LI has been mixed [6,13,21]. Environmental contexts can also change dramatically for young people from adolescence to adulthood. For a number of individuals with LI, compulsory schooling entails difficulties with academic subjects [22] and social difficulties with peer interactions [23]. These factors are likely to increase stress [24] and possibly contribute to the development of symptoms of depression and anxiety [25]. It is not clear whether the type of school attended at 16 years of age affects emotional health. Although special schools may be placements that provide more support, young people at the end of compulsory education attending specialist placements may experience additional stigma. Young people may feel resentful and anxious about the perceived lack of progression to independence and have limited opportunity for typical peer friendships [26]. The end of compulsory education, however, can bring changes in educational and social contexts and opportunities for individuals with LI to pursue their interests via, for example, vocational options offered at college. Taking up such opportunities can lead to an increase in self-esteem and more positive experiences [27]. This, in turn, may mitigate symptoms of depression and anxiety. Indeed Wadman and colleagues [18] found a decrease in depression (but not anxiety) in the period following compulsory education in adolescents with LI. In adulthood, employment pressures are likely to increase for individuals with LI. Education and training do not always translate into employment for these young people [15] and occupational status is strongly related to wellbeing and mental health [28]. On this basis, a re-emergence of depressive symptoms in individuals with LI in adulthood is likely.

It is important to note that although some of the aforementioned environmental changes have been discussed in relation to LI and mental health (e.g. [17]) they are as yet to be investigated directly. This study aims to fill this gap. We use growth curve modelling to determine vulnerabilities to depression and anxiety symptoms from adolescence to adulthood in a prospective longitudinal investigation of children with LI who were attending language units when they were 7 years of age. In the light of previous evidence that individuals with this disorder do experience higher levels of anxiety than do their typically developing peers, we expected to find high levels of anxiety symptoms continuing into early adulthood. Given previous evidence of a reduction in hitherto high symptoms of depression in those with LI at around 16–17 years of age, we sought to determine whether or not this improvement was enduring; a strong possibility was that environmental adversity, such as poor employment circumstances, could impact negatively. In addition to examining verbal and nonverbal skills, we developed a time-varying variable to investigate how transitions from school to employment between adolescence and early adulthood relate to patterns of depression and anxiety symptoms during the same developmental period. Given the widely reported finding that females are significantly more likely to develop depression and anxiety than males [29,30] we investigate also whether the pathways observed differ by gender.

## Method

### Participants

Participants were recruited as part of a large-scale longitudinal research programme which began when the children with LI were 7 years of age [31,32]. At 16 years of age, a typically developing group of young people was recruited as a comparison sample.

#### Young people with LI

The initial cohort of 242 children with LI originally consisted of 186 boys (77%) and 56 girls (23%), and was recruited from 118 language units across England. They represented a random sample of 50% of all 7-year olds attending language units. Language units are specialist resource classes for children who have been identified with primary language difficulties, which are attached to regular schools. The language profiles of the children at recruitment indicated mostly mixed Expressive-Receptive difficulties (53%), and Expressive difficulties only (38%). The remaining children had poor receptive language scores and social communication difficulties. During adolescence, individuals in this group took part in follow-up stages at age 16 (N = 139), age 17 (N = 90) and age 24 (N = 84). Although some attrition occurred over this time, this was partly due to funding constraints/ sub-sampling at follow-up stages of the study at 17 years of age. In addition, some participants who had taken part at age 17 were not traced at age 24 (N = 27, these individuals had data available from 16 and 17 years), and not all of those taking part at age 24 took part at age 17 (N = 21 came back into the study and thus have data at 16 and 24 years). There were no significant differences in receptive or expressive language nor nonverbal IQ (NVIQ) at age 7 between those who participated at age 24 and those who did not (all *p* values >0.2). Attrition was higher for males (60%) compared to females (41%) (*χ*^*2*^ (1) = 7.5, *p* = .006) but the proportion of males (67%) was not significantly different from the age matched peer group (56%; Fishers exact p = 0.16). Participants were included in the study if data was available for at least two of the three time points (16, 17, 24 years), resulting in 107 participants with LI (74 males, 33 females) for the growth curve analysis. In total 59 (55%) of these had data at all three time points, whilst the remainder had 2 data points available (see breakdown above).

#### Age-matched peers (AMP)

The comparison group comprised 99 age-matched peers (AMP; 58 males, 41females) with data for at least two of the three time points for use in the growth curve analysis. This group was recruited to the study aged 16, wherever possible from the same schools as the young people with LI. Thus no early developmental information about language ability at 7 years of age is available. As with the LI group, participation varied at age 16 (N = 121), age 17 (N = 90) and age 24 (N = 66). Some participants had data from age 16 and age 17 (N = 33) and some had data from age 16 and age 24 (N = 10) whilst others had all three data points available (N = 56; 56%). These participants had no history of special educational needs nor speech and language therapy provision. Groups did not differ on age, gender, household income at age 16 (*p* = .80) nor personal income at age 24 (*p* = .40). As expected, language and NVIQ profiles were different for the groups at each time point (Table 1).

**Table 1:** Participant language and NVIQ profiles.

	T1—Age 16		T2—Age 17		T3—Age 24	
	LI	AMP	LI	AMP	LI	AMP
Expressive Language	73.3	98.9	67.0	96.3	70.8	97.7
	(10.2)	(14.7)	(14.4)	(14.0)	(15.7)	(16.3)
Receptive Language	84.6	101.4	75.9	100.3	84.3	105.9
	(17.7)	(13.1)	(18.0)	(11.4)	(18.6)	(9.2)
Nonverbal IQ	85.7	102.1	93.4	106.6	98.9	113.2
	(19.4)	(15.0)	(16.4)	(10.8)	(16.2)	(10.9)

Mean standardised scores where the population mean is 100 (standard deviations scores where the population SD is 15).

### Measures

For anxiety symptoms, the self-report version of the Child Manifest Anxiety Scale—Revised (CMAS-R [33]) was completed at each time point. This is a 28-item questionnaire designed to measure anxiety symptoms in young people aged 6–19 years. Respondents are required to say whether statements are ‘true’ or ‘not true’ for the previous 3 months. The threshold for clinical-level difficulties on this measure is a score above 18.

Depression symptoms were assessed using a self-report version of the Short Form Moods and Feelings Questionnaire (SMFQ [34]), a 13 item questionnaire designed to measure depressed mood in young people aged 8–17. Respondents are required to say whether statements about their feelings were ‘definitely true’ ‘somewhat true’ or ‘not true’ over the previous three months. Both these scales have been used in studies involving young adults and were deemed to remain appropriate for our participants at age 24 years (e.g. [35]). The threshold for clinical-level difficulties on this measure is a score above 7.

For language abilities, the Clinical Evaluation of Language Fundamentals (CELF-R [36] at age 16, CELF-4 UK [37] at ages 17 and 24) was used. To afford measurement continuity the CELF-4 UK was deemed the best fit assessment for our cohort at 24 years of age (neither group reached ceiling levels on this assessment, which is normed up to age 21 years 11 months). The Word Classes subscale for receptive language and Recalling Sentences subscale for expressive language were used at all three time points.

For nonverbal skills, the Wechsler Intelligence Scale for Children (WISC-III [38]) was used at 16 years and the Wechsler Abbreviated Scale of Intelligence (WASI [39]) was used at 17 and 24 years.

### Ethics and procedure

Ethical approval was obtained from The University of Manchester Research Ethics Committee, UK. Written informed consent was obtained from parents or guardians on behalf of the participants enrolled in the study under the age of 18 years. Written informed consent was obtained from the participants themselves at or over the age of 18 years. The participants were interviewed face-to-face at their school or home on the measures described above as part of a wider battery. Interviews took place in a quiet room, wherever possible with only the participant and a trained researcher present. During the interview, the items were read aloud to the participants. The items and response options were also presented visually to ensure comprehension.

### Statistical analysis

A 3-way ANOVA approach was used in the first instance for ease of understanding and interpretation. We report Wilks Lambda statistics because Mauchly’s Test for Sphericity was significant in all cases [40]. However, we are aware that the lack of sphericity combined with incomplete data in places mean that these ANOVAs are likely to underestimate longitudinal effects in this dataset [41]. Thus, to confirm these findings, targeted linear mixed (growth curve) modelling (LMM) was used as this approach affords modelling accounting for attrition across time. A mixed effects model with a maximum likelihood (ML) estimator was used. This allowed for the intercept (depression or anxiety symptoms at baseline) and slope (the rate of change) to vary across individuals. That is, we allowed for starting values of depression or anxiety to vary between individuals and also for individuals to change at a different rate over time. Models were run using the “xtmixed” command. The random part of all models included participant ID and a first order polynomial (time). Figures reported are unstandardized Beta values with 95% confidence intervals. We acknowledge that the LMM analysis makes different assumptions about the correlation and homoscedasticity of the data and also different assumptions as to missing data. However we have included both the ANOVA and the LMM analyses to demonstrate the robustness of the findings. We are also aware that these two methods may be familiar to different audiences and we thought that providing both ANOVA and LMM results would make the findings as accessible as possible.

In addition to the key outcome measures, for the LI group only, concurrent language and IQ scores taken at each time point were regressed onto depression at each age. We also developed a time-varying variable to capture educational and employment transitions of young people with LI at age 16, 17 and 24 years (referred to as the ‘Transition’ variable for ease). The items included in this variable are shown in Table 2, and were binary coded as 0 for more mainstream (1 for less mainstream situations) and were then used as a within-subject profile of transition for each participant allowing us to look at variation in circumstance across time. Each participant was therefore given a grouping classification for transition (000,001,010,100,011,110 or 111). This factor was then used as an independent variable in the modelling. Note that the term ‘Time-varying’ does not suggest time-point as a variable. The variable is included in the model as a single variable, but the value of that variable is not constant over time, and may change from one assessment to the next assessment for any particular individual. It is therefore used as a categorical predictor and is not modelled. Since it is included as a single predictor, without any interaction with time, a single time-constant coefficient is estimated, which is what we report in the results

**Table 2:** Time-varying ‘transition’ variable.

Less mainstream situation (more mainstream)	LI	
	N; %	(N; %)
Special Unit/Special School at 16 (vs mainstream school)	29; 27%	(78; 73%)
Staying in school at 17 (vs college/work)	22; 25%	(67; 75%)
Unemployment or part-time employment at 24 (vs full-time employment/education)	35; 48%	(38; 52%)

All statistical analyses were conducted in SPSS v 22.0 [42] or Stata/SE 13.1 [43]. A two-tailed significance level of *p* = .05 was used unless otherwise specified. Different statistical analyses involve different numbers of participants depending on whether data at all 3 time points (3-way ANOVA) or 2 out of 3 time points (growth curves) were required.

## Results

### Group and gender differences

Descriptive statistics, including the percentage of individuals above the clinical threshold for anxiety and depression symptoms, are shown in Table 3.

**Table 3:** Depression and anxiety symptoms as a function of age, gender and language status.

	Depression (SMFQ)			Anxiety (CMAS-R)		
	Mean (SD)	Mean Diff [95% CI]	% Above Clinical Threshold (n = number)	Mean (SD)	Mean Diff [95% CI]	% Above Clinical Threshold (n = number)
**Age 16**						
LI Male	6.1 (4.7)	-1.3 [-3.5, 1.0]	36.5% (n = 27)	10.1 (6.2)	-1.0 [-3.6, 1.6]	13.5% (n = 10)
LI Female	7.4 (6.7)		42.4% (n = 14)	11.0 (6.6)		15.2% (n = 5)
AMP Male	3.1 (3.8)	-2.3 [-4.0, -0.6]	8.6% (n = 5)	5.8 (4.0)	-2.3 [-4.3, -0.4]	0% (n = 0)
AMP Female	5.4 (4.9)		24.4% (n = 10)	8.1 (5.8)		7.3% (n = 3)
LI Overall	6.5 (5.4)	2.5 [1.1, 3.8]	38.3% (n = 41)	10.4 (6.3)	3.6 [2.0, 5.1]	14.0% (n = 15)
AMP Overall	4.1 (4.4)		15.2% (n = 15)	6.8 (5.0)		3.0% (n = 3)
**Age 17**						
LI Male	5.0 (4.5)	-0.5 [-2.6, 1.5]	18.0% (n = 11)	9.0 (6.2)	-1.9 [-4.8, 1.1]	8.2% (n = 5)
LI Female	5.6 (4.7)		25.0% (n = 7)	10.9 (7.1)		17.9% (n = 5)
AMP Male	3.5 (2.9)	-2.1 [-3.7, -0.5]	13.2% (n = 7)	5.8 (4.1)	-3.3 [-5.3, -1.3]	0% (n = 0)
AMP Female	5.5 (4.7)		24.3% (n = 9)	9.2 (5.5)		2.7% (n = 1)
LI Overall	5.2 (4.3)	0.9 [-0.3, 2.1]	20.2% (n = 18)	9.6 (6.5)	2.4 [0.7, 4.1]	11.2% (n = 10)
AMP Overall	4.3 (3.9)		17.8% (n = 16)	7.2 (5.0)		1.1% (n = 1)
**Age 24**						
LI Male	6.4 (5.5)	-1.0 [-3.6, 1.7]	30.2% (n = 16)	9.5 (6.2)	-2.2 [-5.0, 0.6]	11.3% (n = 6)
LI Female	7.3 (5.8)		40.7% (n = 11)	11.7 (5.3)		7.4% (n = 2)
AMP Male	3.4 (3.1)	-2.6 [-4.6. -0.5]	12.8% (n = 5)	5.9 (5.0)	-2.0 [-4.8, 0.9]	2.6% (n = 1)
AMP Female	6.0 (5.4)		25.9% (n = 7)	7.9 (6.6)		7.4% (n = 2)
LI Overall	6.7 (5.6)	2.2 [0.5, 3.8]	33.8% (n = 27)	10.3 (6.0)	3.6 [1.7, 5.5]	10.0% (n = 8)
AMP Overall	4.5 (4.3)		18.2% (n = 12)	6.7 (5.7)		4.5% (n = 3)

Clinical threshold scores: Depression >7; Anxiety >18.

Anxiety and depression scores correlated with each other highly at each time point (Spearman’s *r* = .7 to .8), and within-anxiety and within-depression moderately across time points (*r* = .3 to .6). A similar pattern was observed between the different language and nonverbal measures. In contrast, associations across language/nonverbal measures and depression/anxiety measures were weaker overall (*r* = .1 to .4).

### Anxiety and depression, group and gender

**Fig 1:**
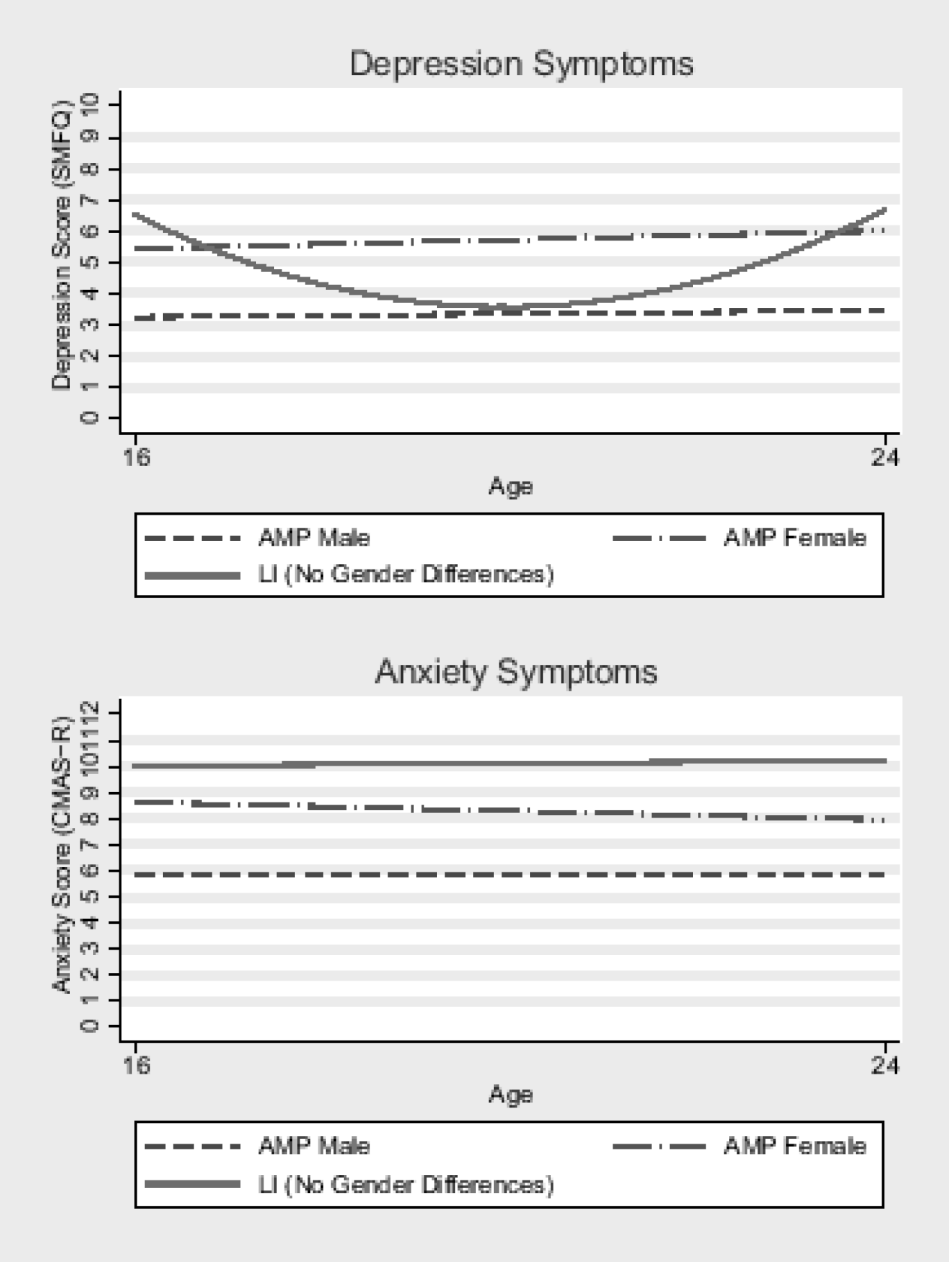
Growth curve models of change in depression and anxiety. Unconditional coefficients for the depression models were as follows: (a) for the participants with LI, linear time (β-1.3 [-2.5, -0.1], p = .032) and quadratic time (β 0.2 [0.0, 0.4], p = .025) were both significant; (b) for the AMP group, neither linear time (β 0.3 [-0.7, 1.2], p = .559) or quadratic time (β -0.0 [-0.2, 0.1], p = .650) were significant.

A 3-way ANOVA was used to explore patterns of change in those with data at all 3 time points. Separate models were run for depression and anxiety. Group (LI or AMP) was a significant predictor of both anxiety (*F*(1,115) = 14.4, *p* < .001) and depression (*F*(1,115) = 8.6, *p* = .004). There was no main effect of Time on anxiety symptoms (*F*(2,114) = 7.5; *p* = .48) or on depression score (*F*(2,114) = 3.2; *p* = .73). For anxiety this was because scores stayed stable and high for the LI group, and stable but low for the AMP group and there was no Group x Time interaction (*F*(2,114) = 1.88; *p* = .16). These findings are illustrated in Fig 1 (bottom panel).

However, for depression, this lack of main effect masked an interaction between Group x Time (*F*(2,114) = 4.5, *p* = .013). There was also a main effect of Gender on anxiety (F (1,115) = 8.0, *p* = .006) and depression (*F*(1,115) = 7.7, *p* = .007). There was no interaction between Gender and Group for anxiety (*F* (1,115) = .01, *p* = .91) or depression (*F* (1,115) = .41, *p* = .52); nor between Gender and Time for anxiety (*F*(2,114) = 1.47, *p* = .23) or depression (*F*(2,114) = .01, *p* = .99). Finally there was also no 3-way interaction (Group x Time x Gender) for anxiety (*F*(2,114) = .55, *p* = .58) or depression (*F*(2,114) = .01, *p* = .99).

### Growth curve models for depression: Groups separate

Given the longitudinal nature of the data and subsequent attrition in our sample, we then sought to confirm the significant interaction between Group x Time for depression using growth curve models. As mentioned before, growth curve models make different assumptions about the correlation and homescedasticity but also, importantly, different assumptions about missing data. Although there is debate about whether three time points are ideal for this analysis, in this instance it allowed us to examine whether the different patterns of change were robust when missing data were modelled. Since we used depression scores as the outcome, all coefficients reported below are group differences or regression coefficients (estimated in a mixed effects context).

Growth curve models were run separately for LI and AMP for depression using participants who had data from at least 2/3 time points available. Predictors were linear time, quadratic time, and gender. For LI participants, linear (*β*-1.3 [-2.6, -0.1], *p* = .031) and quadratic time (*β* 0.1 [0.0, 0.4], *p* = .025) were significant predictors of depression. Gender was not a significant predictor *β* 0.8 [-0.9, 2.5], *p* = .332). For the AMP group, Time was not a significant predictor of depression (linear: *β* 0.3 [-0.7, 1.2], *p* = .555, quadratic: *β* 0.0 [-1.2, 0.1], *p* = .646) but Gender was (*β* 2.3 [1.0, 3.6], *p* = .001) with females showing more depression symptoms than males. Fig 1 gives an indicative illustration of data modelled that shows the patterns observed for depression (top panel, also see legend for information on the unconditional coefficients).

### Lack of associations between depression and verbal and nonverbal kills

Next, because depression in the LI group was of most interest, we tested whether the language and IQ variables predicted this outcome for the LI group only at each of the three time-points separately. None of the predictors were significant: receptive language (16 years: *β* 0.0 [-0.1, 0.0] *p* = .234; 17 years: *β* -0.0 [-0.1, 0.0] *p* = .588; 24 years: *β* 0.0 [-0.0, 0.1] *p* = .778), expressive language (16 years: *β* 0.0 [-0.1, 0.0] *p* = .342; 17 years: *β* -0.0 [-0.1, 0.0] *p* = .404; 24 years: *β* -0.0 [-0.1, 0.0] *p* = .569), and NVIQ (16 years: *β* 0.0 [-0.0, 0.0] *p* = .818; 17 years: *β* -0.0 [-0.1, 0.0] *p* = .350; 24 years: *β* -0.0 [-0.1, 0.0], *p* = .414).

### Transition variable predicts changes in depression in the LI group

The time-varying Transition variable was a significant predictor of change in depression (*β* 1.6 [0.3, 2.8], *p* = .013). Young people with LI who moved out of school (regardless of school type) into college at age 17 and then experienced difficulties gaining full-time employment were more likely to experience the decrease-rise pattern in depression symptoms. A total of 25 participants with LI (47%) showed this pattern. Anxiety symptoms were stable, thus, as expected Transition was not associated with the LI anxiety pathway over time (*β* 1.1 [-0.2, 2.4], *p* = .105).

## Discussion

This study revealed differences in the depression and anxiety pathways of young people with LI from adolescence to adulthood. On the one hand, anxiety symptoms stay stable across time for both groups, the LI group experiencing higher levels of anxiety than their peers from adolescence to adulthood. On the other hand, depression shows a more complex picture, with LI participants experiencing a lessening of symptoms at 17, which is not maintained in adulthood. This picture is mirrored in the number of individuals scoring above clinical thresholds on the measures: more than a third of young people with LI fall into this higher risk group at 16 and 24 years of age, compared to 15–18% of typically developing individuals. Furthermore, the change in depression over time is associated with a particular pattern of changing experience—one in which the pressures of compulsory education are alleviated by more choice-driven college attendance or work experience, only to rise again as employment difficulties become more apparent in adulthood.

These findings support the smaller scale research carried out by Rutter and colleagues (e.g. [5]) and that of Beitchman’s team (e.g. [8,44]) who report higher mental health risk, particularly anxiety, in teenagers and young adults with LI. This longitudinal investigation further specifies that, for anxiety, symptoms remain stable from adolescence to adulthood. In contrast, for depression, the change is complex and non-linear for those with LI. This is not the case for age-matched peers where both depression and anxiety symptoms are stable. Neither language nor nonverbal abilities were significant associates of the depression observed for LI, nor of levels of anxiety at any time point. This is in keeping with previous research on this cohort which showed that, while some weak relationships existed between early language and mental health, this is not an important predictor of depression and anxiety outcomes *per se* [13,18]. Whilst NVIQ was different between the groups and had lowered over time [45], NVIQ was not a predictor of outcome. On the other hand, our analysis does indicate that environmental factors interact with mood vulnerability in this group. Our time-varying variable suggests that different patterns of school and employment transition relate more closely to depression symptoms than language or IQ. In particular, young people moving from school provision into college, who later find themselves without full time employment show a pattern of fluctuating symptoms. Whilst our data does not conclusively speak to why this may be the case, it has been suggested that young people with LI may be more satisfied with lower formal educational outcomes [46] and that a more central construct is peer relationships and friendships [26] which college attendance may have afforded. Because the AMP group showed a stable pattern of depression, we did not further investigate the effect of employment on emotional health more generally. A link between depression and employment status has been reported in the general population, however it should be noted that the direction of association is not clear with suggestion that emotional health influences employment status rather than vice versa [47]. The overall unemployment rate for our AMP group was 7% with a further 19% in part-time work which is lower than the rates for young adults with LI (48%; see Table 2). Future research is needed to examine whether differences in employment across groups could be a cause or an outcome of different patterns of emotional health over time. Those with LI also appear to carry a larger burden of anxiety regardless of these transitions or the severity of their LI (recall that language performance *per se* did not predict anxiety). These results are consistent with other analyses of mental health and language in individuals with LI at different ages [9,20]. They suggest that differences in depression and anxiety symptoms may be part of an inherent co-morbidity that, at least in the case of depression, interacts with environmental factors. It is of course extremely difficult to disentangle the issue of aetiology and phenotype, but it is worth noting that there are alternative explanations to a psychosocial model in which the experience of living with a language difficulty leads directly to emotional health symptoms. This is the first study to examine mental health changes from adolescence to adulthood in LI in relation to *changes* in environmental contexts. It reveals important information for policy and practice. First, the results suggest that individuals with LI are at increased risk of mental health issues over a prolonged period from adolescence to adulthood and that both males and females are equally vulnerable in this population. Awareness of these links is needed in public health services such as community-based doctors/GP practices, mental health teams and social service providers. To our knowledge, in the UK, mental health service providers do not routinely inquire about individuals’ history of language difficulties. Second, it raises the possibility that young people and adults with LI could benefit from improved mental health, in particular depression symptoms, if education/ employment transitions are managed more effectively, and might avoid the need for later service input. Attention to the educational experiences and school transition plans of individuals with LI could help prevention. For example, school transition plans may need to consider mental health support more explicitly and support workers need to be aware that talking therapies may need to be adjusted to take into consideration language abilities, particularly comprehension. Career/employment advice and support in early adulthood could also help mitigate the development and severity of depression symptoms. Such services are available in adulthood for individuals with intellectual and other disabilities such as autism [48], however individuals with LI do not usually meet criteria for such services. LI is a hidden disability [49]. Individuals with LI do not have any outward sign of their difficulties and can fall between stools in terms of access to support.

Participants with LI in this study were recruited once they were attending language units and therefore represent a group of children with severe and persistent LI. Longitudinal examination of mental health pathways of individuals with less severe childhood LI would be useful. There are indications, for example, that mental health outcomes in adulthood of individuals with mild to moderate childhood LI may be comparable to those expected in the general population [17] and a wider ranging sample may reveal stronger associations between emotional health and language. Aspects of the measurements we used could also be built upon. It is worth acknowledging that our ‘transition’ variable was relatively crude and further studies could include more detailed elements of the transition process. As discussed earlier, it would be interesting to research the reasons for the relationship between life transitions and emotional health. Finally, growth curve modelling enables examination of longitudinal patterns where participant attrition has occurred. However these were used in a confirmatory manner, and we acknowledge that increased assessment points at more evenly spaced intervals should be the aim of future studies. In addition, while we are confident that individuals in our original sample who did not participate into adulthood are no different in the early years to those included within this investigation (at least in terms of language and NVIQ—see method), it is not possible to tell whether the pathways of these individuals would have been qualitatively different. In short, data may not be missing at random, but may represent individuals showing important developmental trends not captured here. Further research is needed to replicate our results that introduce the notion of time-varying factors affecting mental health when vulnerabilities are already present.

Nonetheless, this study into the longitudinal pathways of individuals with LI has helped highlight the long-term risk of anxiety and the complex nature of depressive symptoms in this group.
